# Creatine supplementation plus neuromuscular electrical stimulation improves lower-limb muscle strength and quality of life in hemodialysis men

**DOI:** 10.31744/einstein_journal/2020CE5623

**Published:** 2020-11-18

**Authors:** Ana Clara Barreto Marini, Gustavo Duarte Pimentel

**Affiliations:** 1 Universidade Federal de Goiás GoiâniaGO Brazil Universidade Federal de Goiás, Goiânia, GO, Brazil.

**Figure 1:**
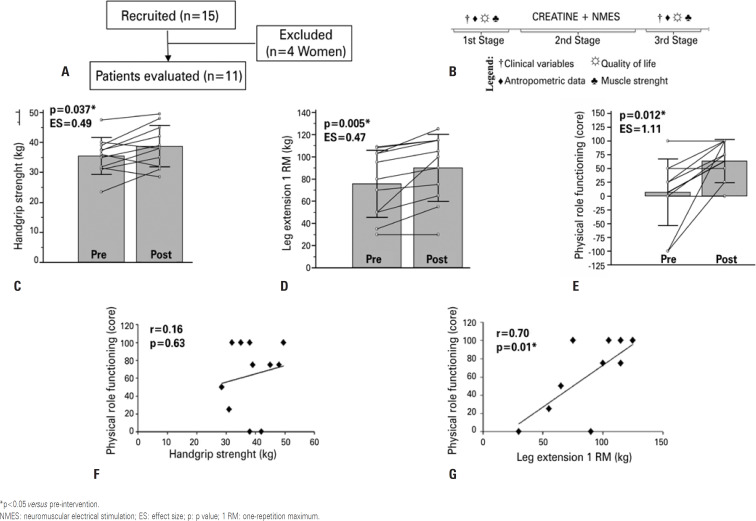
Research methodology and result. (A) participant flowchart (CONSORT). (B) study design. (C) assessment of handgrip strength pre and post intervention. (D) assessment of leg extension with one repetition maximum pre- and post-intervention. (E) assessment of physical role functioning (score) pre- and post-intervention. (F) pearson's correlation of physical role functioning and handgrip strength. (G) pearson's correlation of physical role functioning and one repetition maximum

Dear Editor,

Hemodialysis leads to sarcopenia, a syndrome characterized by the progressive loss of skeletal muscle mass with reduction of physical performance.^(^^1^^)^ Likewise, neuromuscular electrical stimulation (NMES) has been used as strategy for improvement in muscle mass and strength.^(^^2^^,^^3^^)^ We performed a prospective, short-term and single-arm study that evaluated adult men (38.18±12.86 years) undergoing hemodialysis for 40.73±36.98 months ( Table 1 ). Of 15 patients, four women were excluded and 11 men met the inclusion criteria ( Figure 1A ). This study was approved by the Ethical Committee, protocol 1.919.324, CAAE: 51892915.6.0000.5083.

**Table 1:** Baseline features of patients undergoing hemodialysis

Variables		Mean±SD
Age, years		38.18±12.86
Age of diagnosis of CKD, years		35.09±12.54
Length of time on hemodialysis, months		40.73±36.98
Cause of the illness, n=11		
	Hypertension	3
	Diabetes	2
	Glomerulonephritis	3
	Other	3
Smoking (n=11)		
	No	10
	Yes	1
Use of alcohol (n=11)		
	No	5
	Yes	6

CKD: chronic kidney disease; SD: standard deviation.

The interventions were divided into three stages: first week (first stage), consist in the assessment of clinical and anthropometric variables, food intake, muscle strength and quality of life. From the second to the fifth week (second stage) we did the intervention with the creatine supplementation and NMES, and sixth week (third stage), we repeated the first stage. All data were collected within 48 hours after the last supplementation and in the intermediary hemodialysis session ( Figure 1B ). Creatine supplementation was performed as previous described (first week: 20g per day of creatine plus 20g per day of maltodextrin and second to fourth weeks: 5g per day of creatine plus 5g per day of maltodextrin) (Maxtitanium^®^, Supley Laboratório de Alimentos e Suplementos Nutricionais, Matão, SP, Brazil).^(^^4^^)^ Neuromuscular electrical stimulation was done bilaterally at the origin and insertion points of quadriceps or gastrocnemius muscles for 40 minutes during the hemodialysis sessions for three times a week for 1 month.^(^^2^^)^ Body weight (kg), body fat and lean body mass (LBM) were obtained using the dual energy X-ray absorptiometry (DXA) (GE Hologic, Waltham, USA). Height was measured using a portable stadiometer (SECA^®^, Hamburg, Germany) and body mass index (BMI) was calculated based on the body weight and height. Thigh circumference was assessed by using the middle of thigh using an inelastic tape. Handgrip strength was assessed using a hydraulic dynamometer (Takei^®^, Japan) on the non-fistula side. One-repetition maximum (1RM) test was done in sitting position. After three warm-ups with interval of 1 minute of resting each, we performed five attempts to quantify the 1RM. Quality of life was measured using the Medical Outcomes Short-Form Health Survey (SF-36) questionnaire. Food intake assessment was recorded using three 24 hours food recalls, being measured 2 days on weekdays and 1 on the weekends. Food composition was done using the Dietpro^®^ software (Agromídia Softwares, version 5.8, Viçosa, MG, Brazil). The Shapiro-Wilk test was used to test the data normality. Paired Student *t* test was done to assess the difference at pre- versus post-intervention. Relationship between the physical role functioning and handgrip strength and 1RM leg extension was evaluated by the Pearson's correlation. Cohen's d classification was used to verify the effect sizes, such as trivial (d= 0.2), medium (d = 0.5), and large (d≥0.8). Statistical tests were performed using the software MedCalc^®^ Belgium, and the statistical difference was set at 5%.

As a result, we found that body weight, BMI, body fat percentage, LBM and thigh circumference did not change between pre-and post-moment. Handgrip strength and leg extension-1RM was increased in 3.25kg and 14.28kg at the post-intervention (medium effect size), respectively. In addition, the physical role functioning enhanced in 56.82 (large effect size) ( Table 2 ).

**Table 2:** Assessment anthropometric and Medical Outcomes Short-Form Health Survey domains at baseline and after intervention

Variables		Pre	Post	Δ	ES	p value
Anthropometric assessments						
	Body weight, kg	73.28±16.41	73.43±16.66	+0.15	0.009	0.320
	Body mass index, kg/m^2^	24.12±3.74	24.15±3.84	+0.03	0.007	0.570
	Body fat, %	29.46±12.46	29.74±13.22	+0.28	0.021	0.498
	Lean body mass, kg	48.47±7.75	48.23±7.38	-0.24	0.031	0.379
	Thigh circumference, cm	48.26±5.08	48.80±5.75	+0.54	0.099	0.361
Muscle strength						
	Handgrip strength, kg	35.47±6.14	38.72±6.90	+3.25	0.497	0.037 *
	Leg extension-1RM, kg	75.72±30.21	90.00±30.16	+14.28	0.473	0.005 *
Quality of life						
	Vitality	47.27±36.28	63.63±14.33	+16.36	0.593	0.110
	Bodily pain	47.45±36.37	53.90±35.84	+6.45	0.178	0.699
	General health perceptions	33.36±30.31	43.27±15.50	+9.91	0.411	0.279
	Social role functioning	76.13±24.65	70.45±22.55	-5.68	0.240	0.492
	Mental health	72.00±20.70	75.27±13.00	+3.27	0.189	0.515
	Physical functioning †	80 [-50-95]	75 [45-100]	+21.82	0.531	0.173
	Physical role functioning †	100 [-100-100]	100 [0-100]	+56.82	1.116	0.012 *
	Emotional role functioning †	0 [-100-100]	100 [0-100]	+42.42	0.706	0.110

* *t* Student paired;

Δ difference between post and pre;

† data expressed as means and standard deviation or median and minimum and maximum.

ES: effect size; 1RM: one-repetition maximum.

No change in energy, carbohydrate, protein and fat was found ( Table 3 ).

**Table 3:** Assessment food intake at baseline and after intervention

Variables	Pre	Post	Δ	ES	p value
Energy, kcal	1,759.23±801.93	1,508.85±664.44	250.38	0.340	0.239
Carbohydrate, g	225.52±113.98	176.80±73.13	-48.72	0.502	0.145
Protein, g	72.84±46.23	62.56±35.22	-10.28	0.250	0.419
Fat, g	63.04±37.03	61.22±32.49	-1.82	0.052	0.867

Data are expressed as means and standard deviation.

Δ difference between post and pre.

ES: effect size.

We observed a positive correlation between physical role functioning and leg extension-1RM at post-intervention when compared to pre-intervention, but there was no correlation between the physical role functioning and handgrip strength ( Figures 1C to 1G ).

Studies have shown that creatine supplementation and NMES separately can alleviates the LBM loss during hemodialysis.^(^^2^^,^^4^^)^ This is the first study to show that combination of creatine plus NMES enhances the muscle strength and improves the quality of life. In conclusion, creatine supplementation plus NMES has been shown to be a sarcopenia-against important therapy, since it reduces the muscle strength loss and improves quality of life in hemodialysis patients.
