# Supplementary material for: Creatine supplementation plus neuromuscular electrical stimulation improves lower-limb muscle strength and quality of life in hemodialysis men
Source: Einstein (Sao Paulo). 2020 Nov 18;18:eCE5623. doi: 10.31744/einstein_journal/2020CE5623 (PMC7687919; doi:10.31744/einstein_journal/2020CE5623)
Supplement: Translation

# Translation: Suplementação com creatina e estimulação elétrica neuromuscular melhora força muscular de membros inferiores e qualidade de vida em homens em hemodiálise

**Authors:** Ana Clara Barreto Marini, Gustavo Duarte Pimentel

**Affiliations:** 1 GoiâniaGO Brasil Universidade Federal de Goiás, Goiânia, GO, Brasil.

Caro Editor,

A hemodiálise leva à sarcopenia, síndrome caracterizada pela perda muscular esquelética progressiva e por redução do desempenho físico.^(^^1^^)^Da mesma forma, a estimulação elétrica neuromuscular (EENM) tem sido utilizada como estratégia para melhorar a força e a massa muscular.^(^^2^^,^^3^^)^Realizou-se estudo de curto prazo, prospectivo, de braço único, para avaliar homens adultos (38,18±12,86 anos) submetidos à hemodiálise por 40,73±36,98 meses ( Tabela 1 ). De 15 pacientes recrutados, quatro mulheres foram excluídas, e 11 homens preencheram os critérios de inclusão ( Figura 1A ). O estudo foi aprovado pelo Comitê de Ética e Pesquisa, protocolo 1.919.324, CAAE: 51892915.6.0000.5083.

**Tabela 1:** Características de linha de base dos pacientes submetidos àhemodiálise

Variáveis		Media±DP
Idade, anos		38,18±12,86
Idade ao diagnóstico de DRC, anos		35,09±12,54
Duração da hemodiálise, meses		40,73±36,98
Causa da doença (n=11)		
	Hipertensão	3
	Diabetes	2
	Glomerulonefrite	3
	Outros	3
Tabagismo (n=11)		
	Não	10
	Sim	1
Consumo de álcool (n=11)		
	Não	5
	Sim	6

DRC: doença renal crônica; DP: desvio padrão.

**Figura 1:** Metodologia e resultado da pesquisa. (A) quadro de participantes (CONSORT). (B) desenho do estudo. (C) avaliação de força de preensão manual antes e depois da intervenção. (D) avaliação de extensão de perna com uma repetição máxima antes e depois da intervenção. (E) avaliação do funcionamento do papel físico (escore) antes e depois da intervenção. (F) correlação de Pearson do funcionamento do papel físico e força de preensão manual. (G) correlação de Pearson do funcionamento do papel físico e uma repetição máxima

As intervenções foram dividas em três estágios. A primeira semana (estágio 1) consistiu em avaliação de variáveis clínicas, antropométricas, alimentação, força muscular e qualidade de vida. Da segunda a quinta semanas (estágio 2), não houve intervenção de suplementação com creatina e EENM, e, na sexta semana (estágio 3),repetiu-se o primeiro estágio. Todos os dados foram coletados em até 48 horas após a suplementação e sessão de hemodiálise intermediária ( Figura 1B ). A suplementação com creatina foi realizada como descrito (primeira semana com 20g ao dia de creatina, adicionando 20g ao dia de maltodextrina; da segunda a quarta semanas: 5g ao dia de creatina adicionando 5g ao dia de maltodextrina (Maxtitanium^®^, Supley Laboratório de Alimentos e Suplementos Nutricionais, Matão, SP, Brasil).^(^^4^^)^

A estimulação elétrica neuromuscular foi feita bilateralmente na origem e nos pontos de inserção de quadríceps e músculo gastrocnêmio por 40 minutos durante sessões de hemodiálise por três vezes por semana para 1 mês.^(^^2^^)^Peso corporal (kg), gordura corporal e massa magra corporal (MMC) foram obtidos por meio da absorciometria de raios X de dupla energia (DXA; GE Hologic, Waltham, Estados Unidos). A altura foi medida utilizando estadiômetro portátil (SECA^®^, Hamburgo, Alemanha) e o índice de massa corporal (IMC) foi calculado a partir do peso e da altura. A circunferência da coxa foi avaliada no centro da coxa com fita inelástica. A força de preensão manual foi medida com dinamômetro hidráulico (Takei^®^, Japão) do lado não fístula. O teste de uma repetição máxima (1RM) foi realizado com o paciente sentado. Após três aquecimentos com intervalos de 1 minuto de descanso cada, realizaram-se cinco tentativas para quantificar o 1RM. A qualidade de vida foi mensurada por meio de questionário *Medical Outcomes Short-Form Health Survey* (SF-36). A avaliação do consumo de alimento foi gravada utilizando três chamadas para alimentação a cada 24 horas, sendo 2 dias durante a semana e 1 nos fins de semana. A composição dos alimentos foi analisada com o software Dietpro^®^(Agromídia Softwares, versão 5.8, Viçosa, MG, Brasil). O teste de normalidade dos dados foi conduzido por meio do teste de Shapiro-Wilk. Adotou-se o teste *t* pareado de Student para avaliação da diferença no pré e pós-intervenção. O relacionamento entre funcionamento do papel físico, força de preensão manual e 1RM de extensão de perna foi avaliado por meio da correlação de Pearson. A classificação d de Cohen foi aplicada para verificar o tamanho do efeito, tal como trivial (d=0,2), médio (d=0,5) e longo (d≥0,8). Os testes estatísticos foram realizados com o *software* MedCalc^®^Belgium, e a diferença estatística foi estabelecida em 5%.

Como resultado, observou-se que peso corporal, IMC, porcentagem da gordura corporal, MMC e circunferência da coxa não mostraram mudança entre o período pré- e pós-intervenção. A força de preensão manual e extensão de perna no 1RM foi aumentada em 3,25kg e 14,28kg, respectivamente, pós-intervenção (tamanho do efeito médio). Além disso, o funcionamento papel físico melhorou em 56,82 (tamanho do efeito grande) ( Tabela 2 ).

**Tabela 2:** Avaliação antropométrica e domínios segundo o *Medical Outcomes Short-Form Health Survey* na linha de base e pós-intervenção

Variáveis		Pré	Pós	Δ	TE	Valor de p
Avaliações antropométricas						
	Peso corporal, kg	73,28±16,41	73,43±16,66	+0,15	0,009	0,320
	Índice de massa corporal, kg/m^2^	24,12±3,74	24,15±3,84	+0,03	0,007	0,570
	Gordura corporal, %	29,46±12,46	29,74±13,22	+0,28	0,021	0,498
	Massa magra corporal, kg	48,47±7,75	48,23±7,38	-0,24	0,031	0,379
	Circunferência de coxa, cm	48,26±5,08	48,80±5,75	+0,54	0,099	0,361
Força muscular						
	Força de preensão manual, kg	35,47±6,14	38,72±6,90	+3,25	0,497	0,037 *
	Extensão de perna -1RM, kg	75,72±30,21	90,00±30,16	+14,28	0,473	0,005 *
Qualidade de vida						
	Vitalidade	47,27±36,28	63,63±14,33	+16,36	0,593	0,110
	Dor corporal	47,45±36,37	53,90±35,84	+6,45	0,178	0,699
	Percepções de saúde geral	33,36±30,31	43,27±15,50	+9,91	0,411	0,279
	Funcionamento do papel social	76,13±24,65	70,45±22,55	-5,68	0,240	0,492
	Saúde mental	72,00±20,70	75,27±13,00	+3,27	0,189	0,515
	Funcionamento físico †	80 [-50-95]	75 [45-100]	+21,82	0,531	0,173
	Funcionamento do papel físico †	100 [-100-100]	100 [0-100]	+56,82	1,116	0,012 *
	Funcionamento do papel emocional †	0 [-100-100]	100 [0-100]	+42,42	0,706	0,110

* teste *t* pareado de Student;

Δ diferença entre pré e pós-intervenção;

† dados expressos por médias, desvio padrão ou mediana e mínimo e máximo.

TE: tamanho do efeito; 1RM: uma repetição máxima.

Não foi observada mudança na energia, no carboidrato, na proteína e na gordura ( Tabela 3 ).

**Tabela 3:** Avaliação do consumo de alimento em linha de base e após intervenção

Variáveis	Pré	Pós	Δ	TE	Valor de p
Energia, kcal	1.759,23±801,93	1.508,85±664,44	250,38	0,340	0,239
Carboidrato, g	225,52±113,98	176,80±73,13	-48,72	0,502	0,145
Proteína, g	72,84±46,23	62,56±35,22	-10,28	0,250	0,419
Gordura, g	63,04±37,03	61,22±32,49	-1,82	0,052	0,867

Dados expressos por médias e desvio padrão.

Δ diferença entre pré e pós-intervenção.

TE: tamanho do efeito.

Foi observada correlação positiva entre funcionamento do papel físico e extensão de perna 1RM no período pós-intervenção comparado ao pré, contudo não foi observada correlação entre o funcionamento do papel físico e força de preensão manual ( Figuras 1C a 1G ).

Estudos têm mostrado que a suplementação com creatina e EENM separadamente pode aliviar a perda de MMC durante a hemodiálise.^(^^2^^,^^4^^)^Da mesma forma, este é o primeiro estudo a mostrar a combinação de creatina adicionada à melhora da EENM, da força muscular e da qualidade de vida. Em conclusão, a suplementação de creatina adicionada à EENM tem se mostrado uma terapia importante contra a sarcopenia, por demonstrar redução na perda da força muscular e melhora na qualidade de vida em pacientes em hemodiálise.
